# Plant traits and ecosystem effects of clonality: a new research agenda

**DOI:** 10.1093/aob/mcu113

**Published:** 2014-06-19

**Authors:** Johannes H. C. Cornelissen, Yao-Bin Song, Fei-Hai Yu, Ming Dong

**Affiliations:** 1 Systems Ecology, Department of Ecological Science, Faculty of Earth and Life Sciences, VU University, De Boelelaan 1085, 1081 HV Amsterdam, The Netherlands; 2 Key Laboratory of Hangzhou City for Ecosystem Protection and Restoration, College of Life and Environmental Sciences, Hangzhou Normal University, Hangzhou 310036, China; 3 State Key Laboratory of Vegetation and Environmental Change, Institute of Botany, Chinese Academy of Sciences, Beijing 100093, China; 4 School of Nature Conservation, Beijing Forestry University, Beijing 100083, China

**Keywords:** Carbon cycling, clonal plant ecology, effect traits, functional traits, litter decomposition, nutrient, ramet, response–effect trait framework, spacer, spatial heterogeneity, water retention

## Abstract

**Background:**

Clonal plants spread laterally by spacers between their ramets (shoot–root units); these spacers can transport and store resources. While much is known about how clonality promotes plant fitness, we know little about how different clonal plants influence ecosystem functions related to carbon, nutrient and water cycling.

**Approach:**

The response–effect trait framework is used to formulate hypotheses about the impact of clonality on ecosystems. Central to this framework is the degree of correspondence between interspecific variation in clonal ‘response traits’ that promote plant fitness and interspecific variation in ‘effect traits’, which define a plant's potential effect on ecosystem functions. The main example presented to illustrate this concept concerns clonal traits of vascular plant species that determine their lateral extension patterns. In combination with the different degrees of decomposability of litter derived from their spacers, leaves, roots and stems, these clonal traits should determine associated spatial and temporal patterns in soil organic matter accumulation, nutrient availability and water retention.

**Conclusions:**

This review gives some concrete pointers as to how to implement this new research agenda through a combination of (1) standardized screening of predominant species in ecosystems for clonal response traits and for effect traits related to carbon, nutrient and water cycling; (2) analysing the overlap between variation in these response traits and effect traits across species; (3) linking spatial and temporal patterns of clonal species in the field to those for soil properties related to carbon, nutrient and water stocks and dynamics; and (4) studying the effects of biotic interactions and feedbacks between resource heterogeneity and clonality. Linking these to environmental changes may help us to better understand and predict the role of clonal plants in modulating impacts of climate change and human activities on ecosystem functions.

## INTRODUCTION

**Fig. 1.**
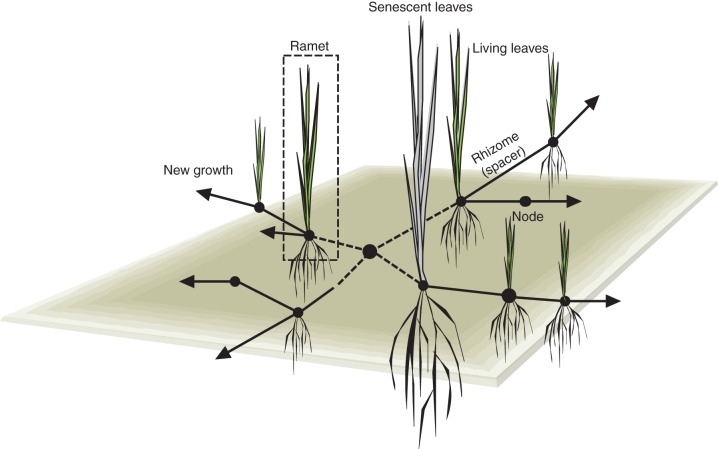
Diagram of a grass showing centrifugal clonal growth, with senescing or dead leaves and rhizomes close to the centre where the mother ramet used to be.

Clonality is an adaptive plant strategy in which ramets (shoot–root units) of the same genetic individual are spaced out and exchange resources through spacers; these spacers can be stolons, rhizomes or roots (de Kroon and van Groenendael, 1997; Xu *et al*., 2012). Clonality is an important way by which plants can reproduce and spread vegetatively (Fig. 1), and clonal structures can also serve as storage organs (Suzuki and Stuefer, 1999; Dong *et al*., 2010). Clonal integration of interconnected ramets has been shown to be advantageous for exploiting resource-rich patches in heterogeneous environments (e.g. de Kroon and van Groenendael, 1997; Jónsdóttir and Watson, 1997; Song *et al*., 2013). Clonality is ubiquitous, especially in environments with abiotic stress (Klimeš *et al*., 1997; Körner, 2003; Ye *et al*., 2014). While clonal plants are very common throughout the monocot clade, the clonal strategy has been adopted by myriad lineages throughout the plant phylogeny (van Groenendael *et al*., 1996; Klimeš *et al*., 1997). However, within the clonal sub-set of the Tree of Life there is also large variation in traits related to clonality, which is the foundation for this review. While clonal traits show substantial intraspecific variation, as related to phenotypic plasticity (Weijschedé *et al*., 2008) and genetic variation (Alpert *et al.*, 2003; D'Hertefeldt *et al*., 2014), the strongest variation is seen among species, and this variation has a strong genetic basis (Pennings and Callaway, 2000; Klimešová *et al.*, 2011; Sammul, 2011).

There is currently a large amount of interest in interspecific variation in functional traits (*sensu* Violle *et al*., 2007), which can be a powerful tool for understanding and predicting (changes in) plant community assembly, functional diversity and biotic interactions under different abiotic and biotic regimes, as well as various key ecosystem functions and services such as productivity, carbon storage, nutrient cycling and water economy (Grime, 2001; Garnier *et al.*, 2004; McGill *et al*., 2006). Measuring and applying variation in clonal traits among species has been increasingly popular for the past few decades (e.g. van Groenendael *et al*., 1996; Klimešová and de Bello, 2009; Herben *et al*., 2014), as such data provide useful information about the performance of clonal species in various habitats varying in resource availability and its spatial heterogeneity, abiotic stress and the presence and abundance of other plant species and other biota (Evette *et al*., 2009; Vermaat, 2009; de Bello *et al*., 2011; Benot *et al*., 2013). Some of the traits commonly studied in such contexts are: spacer length, type and placement; duration of the functional connection between ramets; 2-D spatial pattern of lateral extension; and bud types, placement and densities (van Groenendael *et al*., 1996; Klimeš *et al*., 1997; Klimešová and Klimeš, 2007; Evette *et al.*, 2009). So far the above traits have been studied mostly in connection with their response to environmental factors, by which they promote the fitness of plant individuals and species in their habitats. As such they can all be considered conceptually as ‘response traits’ in the ‘response–effect trait framework’ (Lavorel and Garnier, 2002; Violle *et al*., 2007; Suding *et al*., 2008). The same framework also conceptually defines another type of trait, i.e. ‘effect traits’; these are traits that relate to the potential effect of a species on important ecosystem properties or services, for instance water or carbon storage, productivity, nutrient availability, nectar supply to pollinators and people, and local biodiversity.

Crucial to this framework is how much of the variation in relevant response traits across the species in an ecosystem corresponds to the variation in effect traits of particular interest among the same species. This overlap will determine how different drivers, e.g. landuse or climatic changes, will affect key functions and services through the species composition of the ecosystem (for details of this principle, see Suding *et al*., 2008; Díaz *et al*., 2013; Cornelissen and Makoto, 2014).

**Fig. 2.**
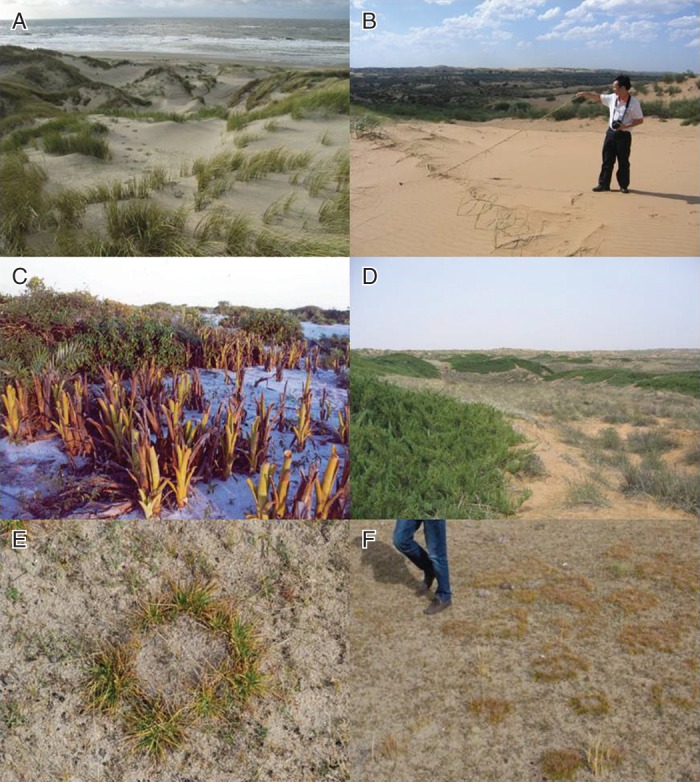
Examples of linear (A–C) and round (D–F) spatial patterns of clonal plants that may affect spatial patterns of soil organic matter, nutrient availability and moisture. (A) *Ammophila arenaria* in Dutch coastal sand dunes; (B) *Psammochloa villosa* on sand dunes in Inner Mongolia, China; (C) *Aechmea nudicaulis* on a sandy beach in Brazil; (D) *Sabina vulgaris* on sand dunes in Inner Mongolia, China; (E, F) *Kobresia humilis* on the shore of Qinghai Lake, Qinghai, China. Photos by the authors, H. de Kroon and M. Sampaio.

It is obvious that clonal plants, with their special structures, control important ecosystem functions; for instance, rhizomes of marram grass (*Ammophila arenaria*) or other monocots help to build and stabilize sand dunes (Fig. 2A). Some empirical studies have found clear effects of clonal integration between ramets on community productivity (Wilsey, 2002; Yu *et al*., 2010; but see Yu *et al*., 2009). There is also some literature on how turf structure of clonal bryophytes controls important functions such as ecosystem hydrology and permafrost maintenance, as detailed with examples in the Supplementary Data. Otherwise, however, there is mostly only anecdotal information about how trait variation among species underpins ecosystem functions related to carbon, nutrient and water cycling. The aim of this review is to define a new approach and research agenda for studying interspecific variation in effect traits of clonal plants related to key ecosystem functions in a concerted manner; and to pinpoint specific traits that bear much promise in this respect. Again, crucial to this research agenda is how much of the variation in key response traits corresponds to the variation in effect traits of particular interest among the same species. This variation can be compared across clonal species or in comparison with non-clonal species. We will put particular emphasis on effect traits that may define variation in plot-scale spatial and temporal heterogeneity of ecosystem functions, as such heterogeneity, especially in soil properties, may be an important determinant of alpha-diversity and species composition of both plants and their associated organisms (Gigon and Leutert, 1996; Grime, 2001; see below).

## CLONAL PLANTS AND SOIL ORGANIC MATTER

Let us now apply the response–effect trait framework to vascular clonal plants. Here we ask the question of how different lateral spatial patterns of clonal extension impact spatial and temporal heterogeneity of carbon, nutrient and water cycling, through variation in the effect traits of different (clonal and other) organs of different species. This is an important question bearing on local-scale (alpha-) diversity and species composition, which are known to be a function (at least partially) of spatial and temporal niche diversity (Gigon and Leutert, 1996; Grime, 2001; Mota de Oliveira *et al*., 2009; de Bello *et al*., 2011). While the response–effect concept, and its consequences for biodiversity, will also apply to some degree to established vegetation later in the succession, early-successional habitats host the most evident examples of how these relationships play out both in theory and in the real world. In order to do so we first have to introduce some basics about the comparative ecology of litter decomposition and its underlying plant traits. There is now a large body of literature showing that variation in leaf (effect) traits of different plant species has strong ‘afterlife’ effects on the decomposition of the litter derived from these leaves (Cornelissen *et al*., 2004). Differences in litter decomposability among species can be tested by incubating multiple species simultaneously in litterbags in a common ‘litterbed’, the latter providing a standardized but relatively natural litter matrix for decomposition (Cornwell *et al*., 2008). From these litterbed studies and other ‘common garden’ studies, we know that relatively tough (high dry matter content), long-lived leaves that are high in lignin and tannins, often acidic and low in base cations and perhaps in nitrogen and phosphorus, tend to be recalcitrant to decomposition compared with juicy (low dry matter content), short-lived, higher pH leaves low in lignin and tannins (Cornelissen and Thompson, 1997; Pérez-Herguindeguy *et al*., 2000; Cornelissen *et al*., 2006; Freschet *et al.,* 2012; Makkonen *et al*., 2012). Recently these leaf-based relationships have been extended to the whole plant. Freschet *et al*. (2012) demonstrated that there was substantial co-ordination of decomposability of different plant organs across many sub-arctic species. At one (slow-turnover) end of the spectrum the species had low decomposability for leaves, fine stems and roots compared with the same organs in other species, while at the other (fast-turnover) end of the spectrum species had relatively high decomposability for the same organs. Relatively low decomposability within each of these plant parts between species could again be linked to high lignin and dry matter content, high tannins, low pH and low nutrient contents.

Now the interesting question arising in the context of clonality is how decomposable dead rhizomes and stolons (Fig. 1) are compared with the other organs of the same species; and how rhizome and stolon decomposition varies among species. Weaver (1947) reported the only study, known to us so far, that compared several grass species for decomposition rates of roots and rhizomes. He found differences in the decomposition rate of roots and rhizomes among species and noted that in some but not all of the species the rhizomes were decomposed faster than the roots. Such comparative information could be important, for example, for predicting the stability and soil formation of sand dunes inhabited by different grass species. It is well known that the rhizomes of some dune grasses such as *A. arenaria* in Europe (Fig. 2A) and *Psammochloa villosa* in China (Fig. 2B) help to build and establish sand dunes during their life time. However, equally important is their function (and that of the roots also still in the sand) after they have died (Fig. 2B). Depending on their structural and chemical effect traits (see above and below), they may decompose fast or slowly, and this is likely to be species dependent. In the case of slow decomposition, they will continue to provide dune stability for much longer than if they decompose fast, providing a long-lasting scaffolding. Also they will help to build up organic matter in a way that will help water retention of the dunes and release nutrients slowly, providing a steady resource supply for other plant species, and other organisms, to establish. Interestingly, very few clonality researchers ever study their plants beyond the life time of their organs (but see Yu *et al*., 2011), even though this is evidently a period of great importance in terms of soil formation and function, and its associated ecosystem services. There are great opportunities here for future research, some of which are related to the consequences of spatial clonal patterns hypothesized below.

## CLONAL TRAITS AS DRIVERS OF SPATIAL HETEROGENEITY OF SOIL RESOURCES

**Fig. 3.**
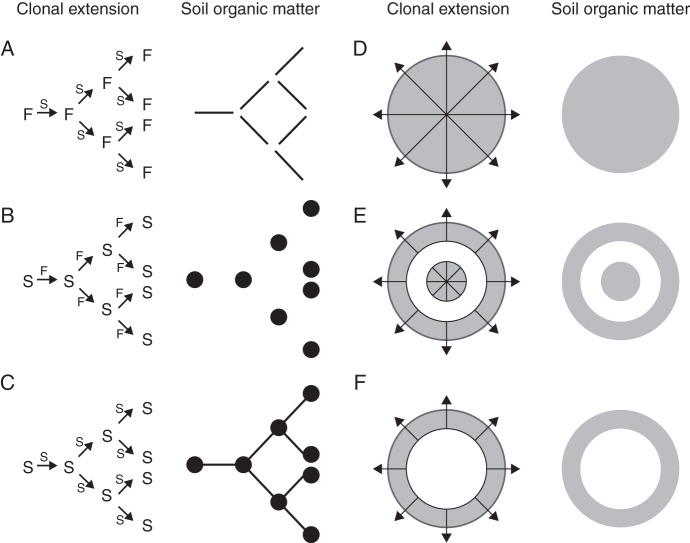
Spatial patterns of clonality and hypothesized organic matter accumulation. For each spatial pattern, the left panel indicates clonal lateral extension and the right panel indicates the pattern of organic matter that could be the longer term consequence of it in terms of soil organic matter. (A) Spacers turn over slowly (S) owing to litter recalcitrance and low decomposability, while the leaves (and roots) of the ramets turn over fast (F) because of high decomposability. Thus only the spacers leave a long-term legacy as linear patterns of soil organic matter accumulation. (B) The opposite pattern where the ramets turn over slowly and the spacers fast, resulting in spotwise patterns of soil organic matter organized in linear configurations. (C) Both spacers and ramets produce recalcitrant litter, resulting in continuous soil organic matter patterning [perhaps exemplified by the bromeliad *Aechmaea nudicaulis* on sandy beaches in coastal Brazil (Fig. 2C)]. (D) Clonal growth pattern where leaves, roots and spacers fill up an entire spot gradually and produce litter of low decomposability and a continuous area of soil organic matter. (E, F) Centrifugal clonal extension where the ramets, with leaves (and roots) of low decomposability, are spread out by spacers, thereby assuming ring shapes, eventually leading to ring-shaped soil organic matter patterns. See the main text for other theoretically possible patterns for clonal and non-clonal plants.

Now, to return to spatial heterogeneity, there is large varation in clonal growth form (Jónsdóttir and Watson, 1997), which should have consequences for spatial patterns of soil organic matter. Let us distinguish two extremes of 2-D lateral extension patterns of clonal plants, a linear and a radial one broadly corresponding to the guerrilla and phalanx strategies (Lovett-Doust, 1981). In the case of a linear pattern, we can distinguish three hypothetical spatial arrangements of organic matter formation in young soils (Fig. 3A–C). They differ in whether the spacers and leaves (and roots), respectively, turn over fast or slowly, as determined by the afterlife effects of their traits on decomposition rates (Freschet *et al*., 2012), some of which have been discussed above. The three scenarios illustrated differ in their spatial distribution pattern of patches of organic matter accumulation. The reference for these three scenarios of clonal plant legacy on linear organic matter pattern would be a clonal species with both short-lived and highly decomposable leaves and spacers, which would not leave much behind in terms of organic matter. Clonal N_2_-fixing species might be a special case of fast-growing plants that do leave a longer term legacy in the soil, e.g. *Trifolium* spp. or other fast-growing legumes with rhizobial symbiosis. Such plants can add significant amounts of new nitrogen to young, nitrogen-poor soils, thereby helping other plants to establish and perhaps indirectly driving directional spatial patterns of soil and vegetation development.

Many other clonal plants show centrifugal radial patterns of lateral extension (Fig. 3D–F). Depending on the clonal traits (e.g. spacer placement angles and lengths) of different species, the lateral extension can lead to broadly circular filled green patches (Fig. 3D) or to ring-shaped ones (Fig. 3E, F), while combinations of linear and radial are also possible in the case of centrifugal linear extension (Fig. 1). It is clear that such special spatial vegetation patterns may be associated with similar patterns of soil organic matter formation. For instance, in the case of the woody gymnosperm *Sabina vulgaris* in Inner Mongolia, China (Fig. 2D), clonal extension leads to very dense green patches consisting of rather tough leaves (the authors' own observations). Like most gymnosperm leaf litter (Cornwell *et al*., 2008), these leaves turn into poorly decomposable litter (G. F. Liu *et al*., unpubl. res.), and the branches, once dead, presumably turn into rather persistent litter as well, as seems to be the rule for gymnosperms (Pietsch *et al*., 2014). We would therefore hypothesize these patches to build up a lot of persistent organic matter, with good water retention also aided by the shaded regime above-ground. In this case there is empirical evidence to support this hypothesis. Ning *et al.* (2013) showed that litter layer thickness, organic matter content, soil water content and soil nitrogen pools were higher within than outside *Sabina* patches. Thus, through a combination of traits related to clonal expansion, and effect traits related to canopy shading and litter decomposability, *Sabina* creates large patches of high dune stability. This stabilizing function of a clonal plant is very important in view of the huge sand and soil erosion and movement problems in northern China, with blinding and damaging sand storms moving into Beijing as one of the expressions of the negative human consequences involved. A contrasting example features *Populus tremuloides*, a strongly clonal tree species, which was shown to have higher leaf litter decomposability than other tree species, especially *Picea mariana*, in its direct surroundings, leading to accelerated nutrient cycling where it occurs (Legare *et al*., 2005). Combined with observations of its centrifugal clonal spread, this species may be hypothesized to accelerate nutrient cycling in somewhat round patches in black spruce forests with strong organic matter accumulation, i.e. the inverted image of Fig. 3D.

It is important to note that all the above patterns are spatially very different from those that may be left by non-clonal plants. Slow-turnover organs of non-clonal plants, and litter from them, may leave small spots of organic matter accumulation in irregular, perhaps even random patterns as determined by seed dispersal and seed rain pattern. On the other hand, fast-turnover organs of non-clonal plants such as short-lived leaves will be highly decomposable and leave no legacy of importance for soil development and biodiversity, as perhaps in the case of the annual *Cakile maritime* in European primary coastal sand dunes. Also, regardless of the spatial patterns, clonality itself is such a major factor in soil development, stability and functioning simply by building integrated networks of spacers and ramets, some of which may persist and impact soil functions long after the life span of these organs.

## CLONALITY EFFECTS ON ECOSYSTEM FUNCTIONS: A RESEARCH AGENDA

### Extended research themes

The above examples merely serve to indicate the potential for ecological clonality and trait research to tackle important new questions related to the impact of clonal plants on ecosystem services and, thereby, on biodiversity. In doing so we have not touched upon several other important aspects of connections between clonality, traits and ecosystem functions for the sake of brevity. Here we briefly mention a few that may be of particular interest.

First, clonal growth patterns as described above may lead not only to spatial patterns of organic matter formation, nutrient enrichment and associated soil functions, but also to patterns of temporal heterogeneity herein. For instance, seasonal patterns of litterfall have recently been shown to be important for the composition and activity of soil microbial communities, with consequences for decomposition and other soil processes (Pearse *et al*., 2013; Thoms and Gleixner, 2013). If, in a clonal context, the fast-turnover leaves of a certain clonal plant species die back and join the soil as litter within the same brief season every year, while rhizomes or other plant parts form litter slowly but steadily throughout the year, then there might be an annual brief season of spatially regular, spot-wise nutrient enrichment. This might lead to simultaneous spatial and temporal heterogeneity of carbon and nutrient dynamics with possible positive effects on alpha-diversity of soil organisms.

**Fig. 4.**
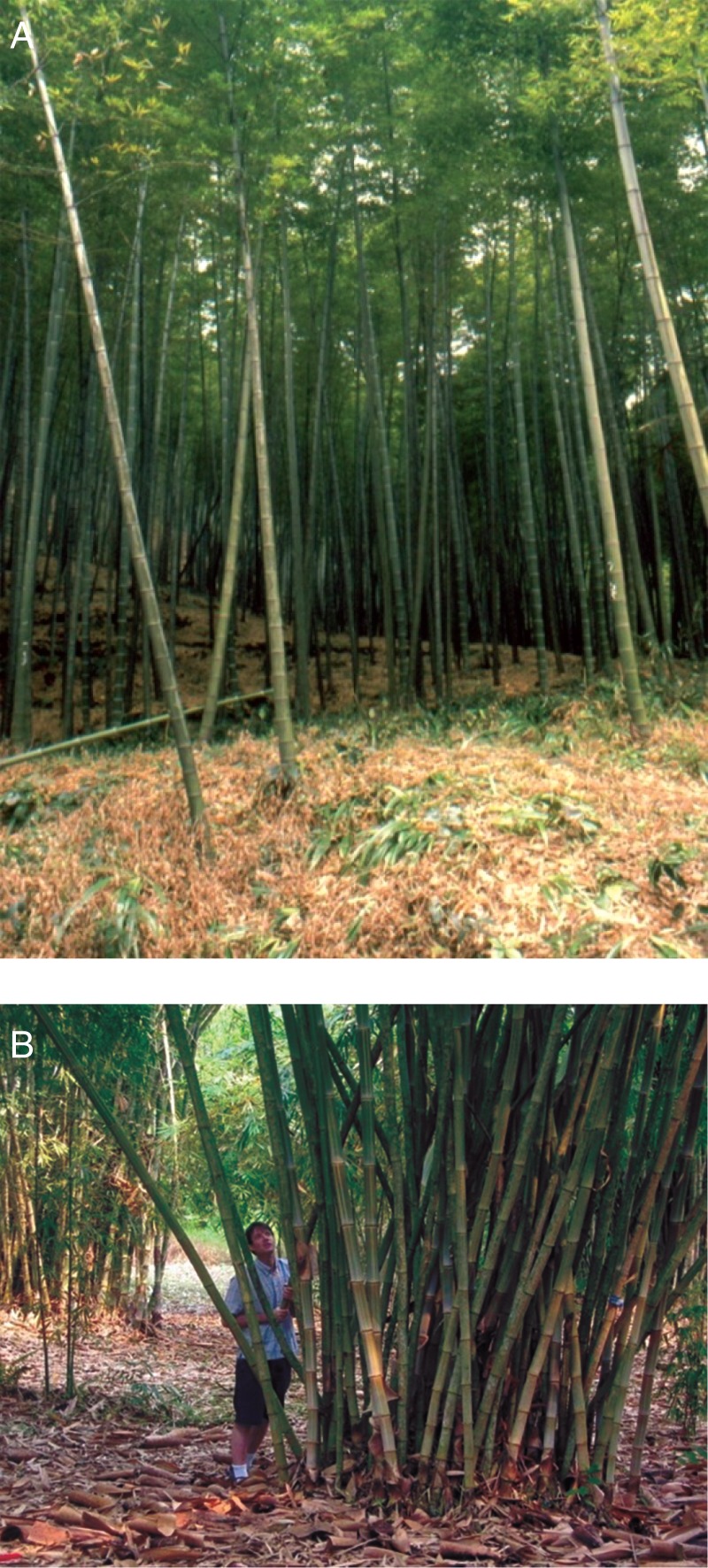
Contrasting clonal bamboo growth forms in China, with implications for spatial and temporal patterns of soil carbon and nutrient dynamics. Bamboo species vary in phalanx vs. guerrilla growth form and degree of woodiness, while some species also show synchronized stem mortality on decadal time scales against leaf turnover on an annual cycle. All this, in combination with large interspecific variation in decomposability for both bamboo leaves and stems (G. F. Liu *et al.*, unpubl. res.), could result in differential effects of different bamboo species on soil carbon and nutrient dynamics in both space and time. (A) *Phyllostachys pubescens* on Mount Jinyun, Chongqing, with guerrilla growth form. (B) *Dendrocalamus* spec. in Xishuangbanna Tropical Botanical garden, with phalanx (clumped) growth form. Photos by J. H. C. Cornelissen.

Secondly, as hinted above, to understand and predict the consequences of clonal plants for soil carbon and nutrient stocks and dynamics, it is important to know both the relative amounts of each plant organ entering the soil as litter, as determined by biomass allocation and organ life span, and the quality and decomposability of that litter (Freschet *et al*., 2013). Together these factors will determine the extent to which a plant species will provide overall positive or negative feedback to ecosystem-scale decomposition rates, or whether the effects of different organs will partly cancel each other out, e.g. if the leaves of a species are more decomposable than those of other species while its fine stems are less decomposable than those of the other species. In a clonal context, biomass allocation to and decomposability of different organs will also affect the spatial and temporal patterns of soil organic matter formation and associated services. The contributions of roots and, in the case of shrubs, trees and bamboos (details in Fig. 4), (fine and coarse) wood to soil function need to be considered in combination with those of leaves and rhizomes, and in their spatial context.

Thirdly, we have so far focused on early-successional ecosystems with very young soils, where effects of clonal plants on ecosystem functions are highly visible. However, the role of clonality in ecosystem functions later in the succession is also of great potential interest, but poorly studied to date, perhaps because of the complex interactions with other environmental variables. In a rare study relevant to these questions, Yu *et al*. (2011) found that *Carex sempervirens* tussocks in a sub-alpine grassland in the Central Alps induced spatial heterogeneity in litter decomposition rates, but they could not detect significant effects of this on other soil properties.

Fourthly, intraspecific trait variation has not been considered here so far, but it can make a significant contribution to clonal trait variation (e.g. Weijschedé *et al*., 2008). For instance, clonal growth form can differ strongly within the grass *Leymus secalinus*, even ranging all the way between phalanx and guerrilla strategy (Ye *et al*., 2006), and such variation may lead to similar heterogeneity of soil function as described above for interspecific trait variation.

Fifthly, ecosystem-level effects of clonal integration are still unclear. While there is large intraspecific and interspecific variation in the degree of clonal integration (Pennings and Callaway, 2000; Alpert *et al*., 2003; D'Hertefeldt *et al*., 2014), how this variation contributes to the variation in productivity and nutrient cycling in ecosystems is virtually unknown (Wilsey, 2000; Yu *et al.*, 2009, 2010). If clonal plants are the dominant plant species in an ecosystem (e.g. grassland and wetland), we can expect that the positive effects of clonal integration on the growth and nutrient status of individual plant species can be translated into positive effects on ecosystem productivity and nutrient cycling. In this context, Magyar *et al*. (2004) presented a very promising model, which incorporates effects of clonal plant activity on spatial patterns of resource availability, and thereby succession. Predictions from models such as this should be combined with empirical studies, e.g. field and greenhouse experiments combining the technique of severing inter-ramet connections and stable isotopes.

Sixthly, biotic interactions may moderate the effects of clonal plants on ecosystem functions. For instance, marram grass (*A. arenaria*), mentioned above as a key dune builder (Fig. 2A), is highly vulnerable to root herbivory by nematodes, populations of which build up over time (van der Putten *et al*., 1993). The grass generally succumbs to this attack and thereby gives way to the next phase of dune succession. Knowing relationships between root and rhizome traits of different plant species and other organisms that depend on them will help us better understand below-ground processes and succession.

Seventhly, the spacers of clonal plants, especially rhizomes, may also play a major role in the formation of soil carbon stocks while still alive. This role is based on the storage function these organs have, for instance for carbohydrates, and this function varies greatly among species (de Kroon and van Groenendael, 1997).

Finally, an intriguing and challenging aspect of clonality on the research agenda should be the feedbacks between environmental heterogeneity and clonal trait variation, as already predicted from the elegant modelling exercises by Magyar *et al*. (2004), mentioned above. A large body of literature has demonstrated the role of spatial clonal extension patterns in accessing soil resources, particularly nutrients but also water. There has been much debate about whether and how clonal plants may actively ‘forage’ for nutrients (de Kroon and Hutchings, 1995; Oborny and Cain, 1997), but the fact is that they are effective at exploiting resource-rich patches within heterogeneous environments (Zhou *et al.*, 2012). Based on the scenarios above (see Fig. 3), we also predict that clonal plants themselves create spatial heterogeneity of resources, for instance through the litter and soil organic matter derived from them. Thus, there may be positive or negative feedback of clonality on resource heterogeneity. For example, we hypothesized above that the clonal bromeliad *Aechmaea nudicaulis* would create spatial heterogeneity in litter deposition and organic matter formation on sandy beaches in Brazil, with consequences for nutrient and water supply. At the same time, Sampaio *et al*. (2004) reported that this same bromeliad shows directional movement in response to resource heterogeneity. This suggests there could be positive feedback of the clonal behaviour of this species on spatial patterns of resource distribution.

### Suggestions for specific analyses

We have identified several themes related to clonal traits and ecosystem functions of particular interest for further investigation. The question is how do we go about this in practice. By combining some of the approaches (and existing data) of previous clonality-related investigations with the screening for effect traits now becoming popular in ecology, we may be able to answer some of the questions emerging from this review. On the response trait side, clonal researchers have already published a lot about responses of clonal plants (*vis-a-vis* non-clonal plants) to resource stress and heterogeneity (e.g. de Kroon and Hutchings, 1995; Song *et al.*, 2013 for reviews), and about trait variation that underpins this response (e.g. Alpert *et al*., 2003). Clonal response traits that are of particular interest in this context, and for which species by trait databases are growing already, include clonal vs. non-clonal habit, clonal type, biomass allocation to clonal and other organs, spacer length, spacer 3-D placement, number of clonal offspring ramets, spacer life span and clonal bud density (Klimeš *et al.*, 1997; Klimešová and de Bello, 2009; Sammul, 2011; Herben *et al*., 2014). At the same time, clonal species can be screened for ‘effect traits’, such as water retention capacity (especially in bryophytes), spacer carbon storage capacity (see above), resource redistribution through clonal integration, and litter decomposability of various organs (including both clonal organs such as rhizomes and stolons and other organs). The litter decomposability data can be obtained from litterbed studies (see above and protocols in Pérez-Harguindeguy *et al*., 2013), but measuring certain structural or chemical effect traits may provide shortcuts informing about decomposability: toughness, dry matter content, lignin content, nitrogen content, base cation content and tissue pH (for protocols, see Pérez-Harguindeguy *et al*., 2013). These data would then help to predict how the composition of clonal (vs. non-clonal) species could control soil functions, and the spatial patterns in these functions. These predictions could then be tested through field sampling of spatial distribution patterns of plant species and measurement of soil properties (including water retention capacity, carbon storage and nutrient availability) associated with them. Such a combination of approaches would enhance our understanding of the mechanisms involved and the predictive power of effects of clonal plants on ecosystems.

## CONCLUSIONS AND OUTLOOK

This conceptual review has highlighted the following new research agenda for linking traits of clonal plants to ecosystem functions.

1. The degree of correlation between clonal response traits and (clonal or other) ecosystem effect traits among species may help us to understand soil stability and carbon, nutrient and water cycling, especially in early-successional but probably also in later-successional environments.
2. Such insights may help us to understand spatial and temporal heterogeneity in these ecosystem functions, especially when species variation in effect traits is linked to clonal response trait variation and actual field distribution patterns of clonal plants.
3. Spatial and temporal heterogeneity in these functions, as influenced by clonality, may in turn support the diversity of other species, another promising field of investigation.
4. A great challenge of particular importance is to understand feedbacks between resource heterogeneity and clonality, as there is mutual causality between them.
5. Linking all these pieces of the big puzzle to actual or predicted environmental changes and population dynamics may help us to understand the role of clonal plants, whether native or invader, in modulating impacts of global change and human activities on ecosystems.

## SUPPLEMENTARY DATA

Supplementary data are available online at www.aob.oxfordjournals.org and consist of detailed examples of clonal bryophytes and their influence on ecosystem water regulation.

Supplementary Data
