# Supplementary material for: Plant traits and ecosystem effects of clonality: a new research agenda
Source: Ann Bot. 2014 Jun 19;114(2):369–76. doi: 10.1093/aob/mcu113 (PMC4111380; doi:10.1093/aob/mcu113)
Supplement: Supplementary Data [file supp_114_2_369__index.html]

Plant traits and ecosystem effects of clonality: a new research agenda — Supplementary Data 

# Plant traits and ecosystem effects of clonality: a new research agenda

## Supplementary Data

Supplementary Data

**Files in this Data Supplement:**

- Supplementary Data - Pdf file
