# Supplementary material for: Plant traits and ecosystem effects of clonality: a new research agenda
Source: Ann Bot. 2014 Jun 19;114(2):369–76. doi: 10.1093/aob/mcu113 (PMC4111380; doi:10.1093/aob/mcu113)
Supplement: Supplementary Data [file supp_mcu113_mcu113supp.pdf]

SUPPLEMENTARY DATA

Clonal bryophytes and water regulation

Clonality of bryophytes in connection with ecosystem water regulation provides a clear example where studying effect trait variation among species may be rewarding. Most bryophytes are highly clonal (Barkman, 1958; During, 1990; Robinson and Miller, 2013); while most species reproduce sexually and this is important for genetic exchange and diversity in the long run, at shorter time scales the importance of vegetative expansion and reproduction seems to predominate. Bryophytes, i.e. mosses, hornworts and liverworts, are well known for their important role in water retention, for instance, as epiphytes on trees (Cornelissen and ter Steege, 1989; van Leerdam *et al*., 1990). Many moss and liverwort genera are known to form extensive cushions in tropical rainforest tree trunks and canopies, together covering much of the surface area of these trees with luxuriant green mass, as a water storage station between precipitation entering the forest and stem flow towards the soil. However, there is great interspecific variation in the position, thickness and lateral extent of bryophyte cushions. Among neotropical epiphytic bryophytes, for example, several *Bazzania* and *Plagiochila* species produce enormous wet cushions on big boles and in tree forks, while some *Frullania* and *Diplasiolejeunea* species form very thin mats intimately pressed onto thin canopy twigs and *Taxithelium planum* and *Symbiezidium transversale* extend horizontal shoots laterally around upright trunks, being specialized at catching stem flow (Cornelissen and ter Steege, 1989; Wolf, 1993). These different growth forms depend on whether and how different individual shoots (ramets) of a genet are connected to form a cushion or turf; which in turn depends greatly on interspecific variation in clonal traits, i.e. the types and lengths of connecting structures as well as their angles, densities and longevities. There is no literature that has quantified such trait variation, or how it relates to cushion structure and its water regulation function, which hampers our understanding and predictive capacity of bryophyte water regulation as dependent on species composition.

Another example comes from the arctic tundra, also known for its abundant bryophyte growth. Here also, the structure of the cushions or turfs of different species regulate ecosystem water retention (Dilks and Proctor, 1979; Glime, 2007), and thereby soil temperature regimes and freeze-thaw cycles (Gornall *et al*., 2007; Soudzilovskaia *et al*., 2013), thereby also being instrumental in the maintenance of permafrost and the long-term storage of frozen organic carbon. Peat mosses, *Sphagnum*, known to be the ultimate ‘sponges’ among the mosses, are very important for these functions, but water regulation varies greatly among *Sphagnum* species differing in turf growth form and density (see Fig. S1). We expect

that this variation in turn depends on variation in their clonal traits, but this has not been reported in the literature – although some of this information may be derived from drawings in identification guides. Perhaps in a less spectacular way, a wide range of non-*Sphagnum* mosses are also important in tundra water regulation. Elumeeva *et al*. (2011) water-saturated both single shoots and substantial, similarly sized turfs of multiple bryophyte species from sub-arctic Sweden in a greenhouse (Fig. S1A) and measured the time to 50 % water loss (t_{50}). Interestingly, the interspecific variation in t_{50} among single shoots across species was not correlated with that in t_{50} of the cushions. The mechanistic explanation for this is that shoot water retention is driven by internal tissue traits such as cell wall thickness, while cushion water retention depends greatly on the retention of external water between shoots as a function of cushion density (Elumeeva *et al*., 2011), which in turn must depend on interspecific variation in the clonal traits mentioned above for epiphytes. Interestingly in this context, Michel *et al*. (2012) demonstrated that the architecture of mixed bryophyte species in cushions (see Fig. S1B), presumably based at least partly on variation in clonal traits of the component species, had non-additive effects on the water retention capacity relative to the predicted retention capacity based on the monocultures of the component species. If we can predict the ecosystem water retention of different bryophytes based on their clonal (and cell-level) traits in the ecosystems where they are crucial to their water economy, this would provide a most useful shortcut in estimating this function based on species composition.

FIG. S1: Cushions of subarctic clonal bryophytes from N Sweden. (A) Monospecific cushions (and a few macrolichens) showing different structures as determined by their clonal traits; (B) Mixed cushions in which non-additive interactions on water retention may occur. Photos by N.A. Soudzilovskaia and J.H.C. Cornelissen.

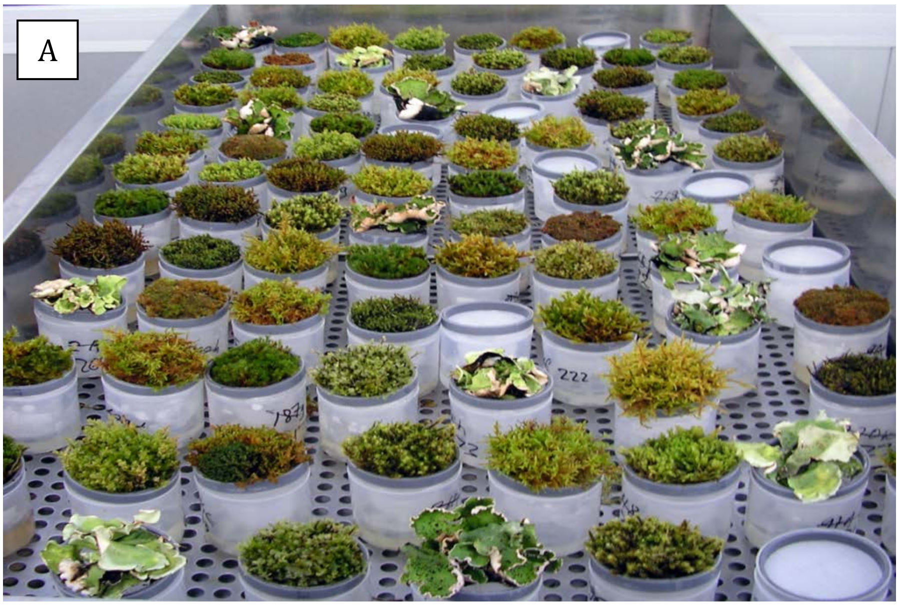

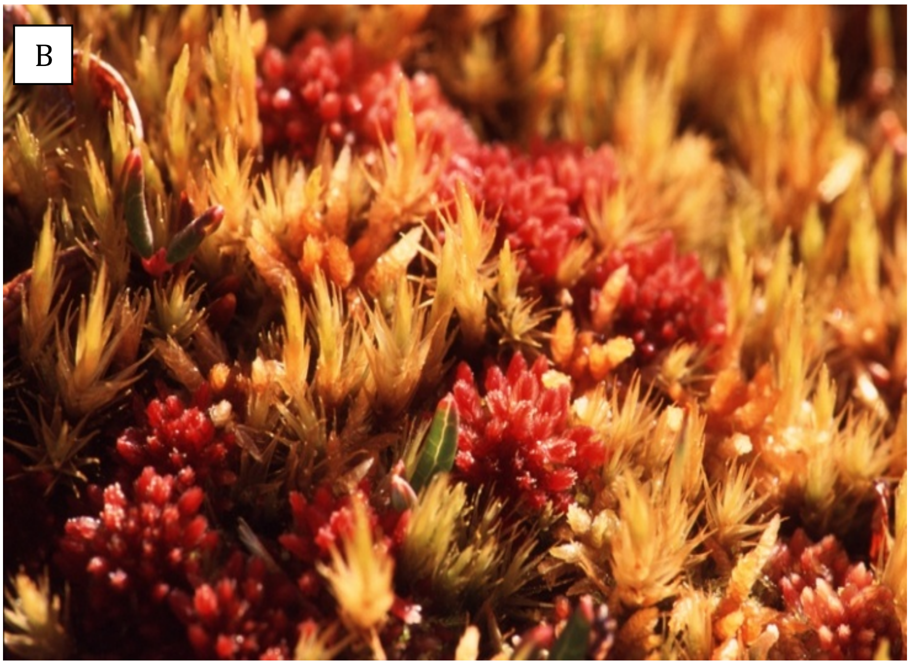

References

Barkman JJ. 1958. *Phytosociology and Ecology of Cryptogamic Epiphytes.* Assen: Van Gorcum.

Cornelissen JHC, ter Steege H. 1989. Distribution and ecology of epiphytic bryophytes and lichens in dry evergreen forest of Guyana. *Journal of Tropical Ecology* **5**: 131-150.

Dilks TJK, Proctor MCF. 1979. Photosynthesis, respiration and water content in bryophytes. *New Phytologist* **82**: 92–114.

During HJ. 1990. Clonal growth patterns among bryophytes. In: van Groenendael J, de Kroon H, eds. *Clonal Growth in Plants: Regulation and Function*. The Hague: SPB Academic Publishing.

Elumeeva TG, Soudzilovskaia NA, During HJ, Cornelissen JHC. 2011. The importance of colony structure versus shoot morphology for the water balance of 22 subarctic bryophyte species. *Journal of Vegetation Science* **22**: 152-164.

Glime, JM. 2007. Bryophyte ecology. Ebook sponsored by Michigan Technological University and the International Association of Bryologists;
available at http://www.bryoecol.mtu.edu/ (accessed 30 April 2014).

Gornall JL, Jonsdottir IS, Woodin SJ, Van der Wal R. 2007. Arctic mosses govern below-ground environment and ecosystem processes. *Oecologia* **153**: 931–941.

Michel P, Lee WG, During HJ, Cornelissen JHC. 2012. Species traits and their non-additive interactions control the water economy of bryophyte cushions. *Journal of Ecology* **100**: 222-231.

Mota de Oliveira S, ter Steege H, Cornelissen JHC, Gradstein SR. 2009. Niche assembly of epiphytic bryophyte communities in the Guyanas: a regional approach. *Journal of Biogeography* **36**: 2076-2084.

Robinson SC, Miller NG. 2013. Bryophyte diversity on Adirondack alpine summits is maintained by dissemination and establishment of vegetative fragments and spores. *Bryologist* **116**: 382-391.

Soudzilovskaia NA, van Bodegom PM, Cornelissen JHC. 2013. Dominant bryophyte control over high-latitude soil temperature fluctuations predicted by heat transfer traits, field moisture regime and laws of thermal insulation. *Functional Ecology* **27**: 1442-1454.

van Leerdam A, Zagt RJ, Veneklaas EJ. 1990. The distribution of epiphyte growth-forms in the canopy of a Colombian cloud-forest. *Vegetatio* **87**: 59-71.

Wolf JHD. 1993. Diversity patterns and biomass of epiphytic bryophytes and lichens along an altitudinal gradient in the northern Andes. *Annals of the Missouri Botanical Garden* **80**: 928-960.
